# Acute pancreatitis and refractory hypercalcemia in the third trimester caused by parathyroid carcinoma

**DOI:** 10.1186/s12884-024-06636-3

**Published:** 2024-07-17

**Authors:** Qingwen Nie, Shunlin Ouyang, Fang He

**Affiliations:** 1 Department of Obstetrics and Gynecology, Guangdong Provincial Key Laboratory of Major Obstetric Diseases; Guangdong Provincial Clinical Research Center for Obstetrics and Gynecology; Guangdong-Hong Kong-Macao Greater Bay Area Higher Education Joint Laboratory of Maternal-Fetal Medicine, the Third Affiliated Hospital of Guangzhou Medical University, Guangzhou, 510150 China; 2 Department of Otolaryngology, the Third Affiliated Hospital of Guangzhou Medical University, Guangzhou, China

**Keywords:** Parathyroid carcinoma, Acute pancreatitis (AP), Hypercalcemia, Primary hyperparathyroidism (PHPT)

## Abstract

**Background:**

Hypercalcemia can be a rare contributor to acute pancreatitis (AP) in pregnancy. This is primarily due to primary hyperparathyroidism (PHPT), resulting from parathyroid carcinoma. We exhibited a case report to analyze the diagnosis and treatment during the onset of hypercalcemia-induced AP.

**Case presentation:**

A 32-year-old primigravida presented with acute pancreatitis near full-term gestation. Following a cesarean delivery, there was a reduction in serum amylase and peripancreatic exudate, but her serum calcium concentrations persistently elevated over 4.0 mmol/L. Interventions to lower the hypercalcemia were only temporarily effective, until a high serum parathyroid hormone (PTH) concentration of 1404 pg/mL was detected. Ultrasound revealed a 31 mm × 24 mm hypoechoic oval nodule in the left lower lobe of the thyroid gland. She underwent a parathyroidectomy, resulting in a dramatic decrease in serum PTH level, from preoperative levels of 2051 pg/mL to 299 pg/mL just 20 minutes after removal. Similarly, her serum calcium declined from 3.82 mmol/L to 1.73 mmol/L within 24 hours postoperatively. The final histopathology suggested parathyroid carcinoma.

**Conclusion:**

When refractory hypercalcemia is present, serum PTH levels should be measured to determine PHPT. Parathyroidectomy is the optimal strategy for alleviating hypercalcemia and clarifying the underlying pathology.

## Introduction

Acute pancreatitis (AP) in pregnancy due to hypercalcemia is uncommon [1]. The mechanisms underlying hypercalcemia-induced AP involve the deposition of calcium in the pancreatic duct and the activation of trypsinogen within the pancreatic parenchyma [2, 3]. Primary hyperparathyroidism (PHPT) is the most frequent causes of hypercalcemia, accounting for more than 90% of all instances [4]. Long-standing overproduction of parathyroid hormone (PTH) and hypercalcemia can lead to progressive damage to vital organs and even mortality [5, 6]. This report presents a sporadic case of AP resulting from parathyroid carcinoma, occurring during near-term gestations. The objective is to enhance the differential diagnosis of parathyroid carcinoma by elucidating the diagnostic and therapeutic procedures employed for this condition.

## Case presentation

**Fig. 1:**
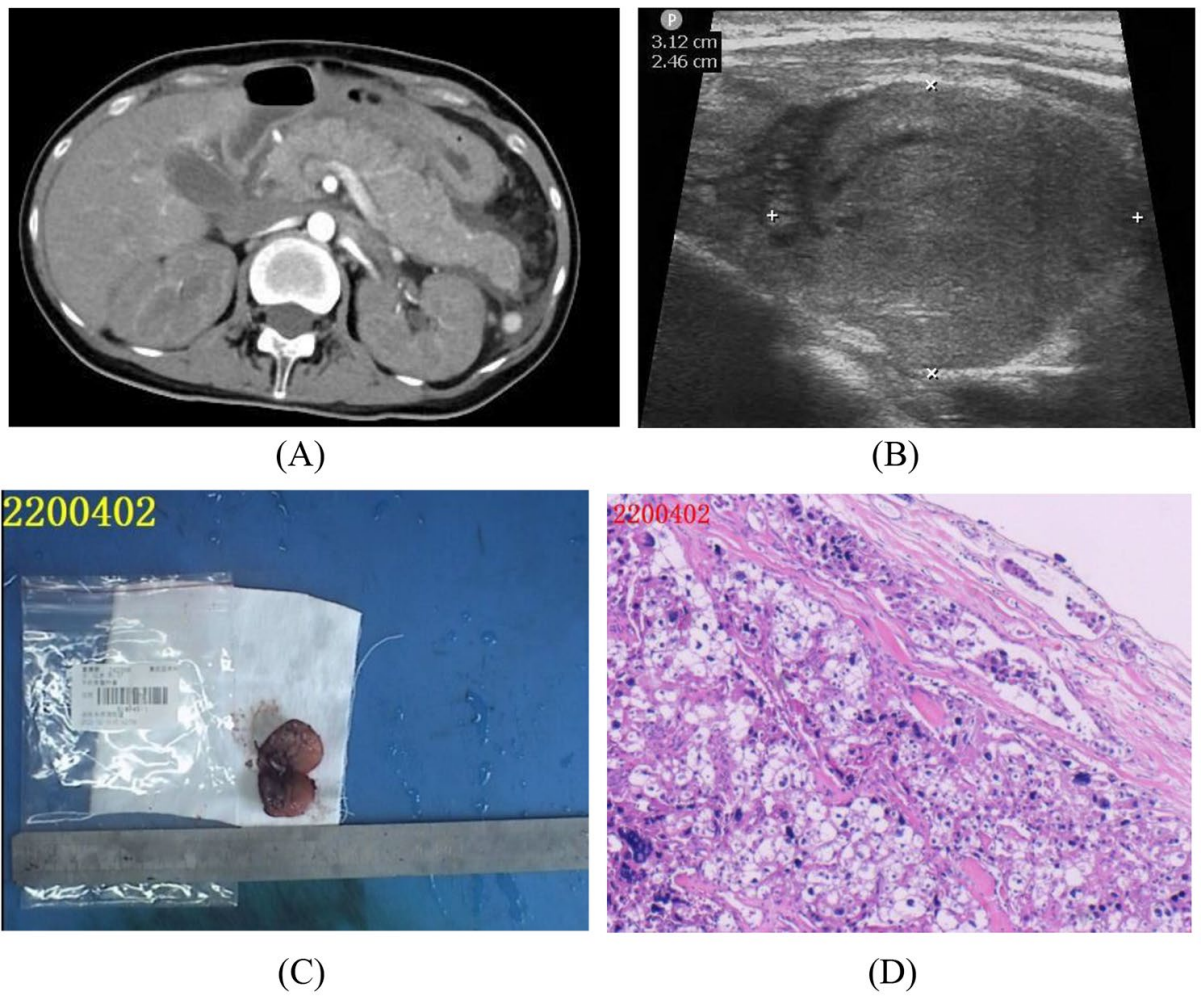
Imaging and pathological findings. (**A**) On Day 1 following admission, CT showed an edematous pancreas. (**B**) A hypoechoic oval nodule with a size of 31 mm × 24 mm in the left lower lobe of the thyroid gland. (C)–(D) Pathology of parathyroid carcinoma. (**C**) Parathyroid gland section profile. (**D**) Hematoxylin-eosin staining (×200): tumor cells invaded the capsule and blood vessels

A 32-year-old primigravida at 36^+ 3^ weeks gestation presented to the emergency department with acute epigastric pain persisting for two days, accompanied by abdominal distension and nausea. Upon the reception, her blood pressure was measured at 135/87 mmHg. Laboratory investigations revealed elevated serum amylase levels of 935U/L (normal range 35–135) and serum creatine of 110µmol/L (normal range 64–104). Abdominal ultrasound confirmed the diagnosis of acute pancreatitis by revealing an edematous pancreas (Fig. 1A). Following admission, an urgent cesarean section was performed, resulting in the successful delivery of a live birth weighting 2470 g with an Apgar score of 10-10-10. The expulsion of the placenta and membranes was complete.

After the cesarean delivery, the patient’s amylase rose to 1501 U/L, and serum calcium concentrations were alarmingly high at 4.06 mmol/L (normal range 2.20–2.55). Due to the severity of conditions, she was transferred to the intensive care unit for further treatment. Although the serum amylase exhibited a stepwise decrease, severe hypercalcemia (over 4.0mmol/L) persisted. Attempts to lower the hypercalcemia with calcitonin and dialysis only provided temporary relief, as serum calcium levels rebounded within one day. Further laboratory investigations found an abnormally high serum PTH concentration of 1404 pg/mL (normal range 10–65). On the fourth postoperative day, ultrasound revealed a 31 mm × 24 mm hypoechoic oval nodule in the left lower lobe of the thyroid gland—suggestive of a parathyroid adenoma (Fig. 1B). A fine needle aspiration (FNA) was performed by an otolaryngologist, and confirmed the tissue as the source of the parathyroid. Immunohistochemistry: CgA(+), CT(-), Cyclin D1(-), Ki-67(< 1%), Syn(-), TTF-1(-). Meanwhile, a contrast-enhanced magnetic resonance imaging (MRI) of the neck soft tissues supported the diagnosis of parathyroid adenoma. After a week of treatment, the serum amylase decreased, and CT images demonstrated a reduction in peripancreatic exudate. Based on a multidisciplinary discussion, the otolaryngologists decided to perform a resection of the left parathyroid adenoma. Serum PTH concentrations dramatically decreased from preoperative levels of 2051 pg/mL to 299 pg/mL at 20 min after the removal of tumor tissue. Serum calcium remained at a concentration of 3.82 mmol/L after the patient returned to the ward, dropped to 1.73 mmol/L within 24 hours postoperatively. Histopathology revealed features of angioinvasion, and immunohistochemical staining exhibited a Ki67 labeling index of 5% (Fig. 1C-D). A PET-CT scan did not reveal any hypermetabolic malignant lesions elsewhere but did identify gallbladder stones, multiple nephrolithiasis, multiple bilateral anterior rib fractures, and systemic osteoporosis. Following the histopathological evaluation confirming parathyroid carcinoma, a remedial surgery including ipsilateral thyroid excision and lymph node dissection was performed. Both intraoperative frozen section analysis and subsequent pathological reports confirmed the presence of a nodular goiter. The patient received intravenous calcium infusion (calcium gluconate 1–2 g/d) combined with oral route postoperatively, until serum calcium levels stabilized. Due to their susceptibility to the hungry bone syndrome after removal of a parathyroid tumor, she was prescribed oral calcium, vitamin D, and osteotriol for three months post-discharge. She has maintained normal serum parathyroid and calcium levels in recent two years.

## Discussion

**Fig. 2:**
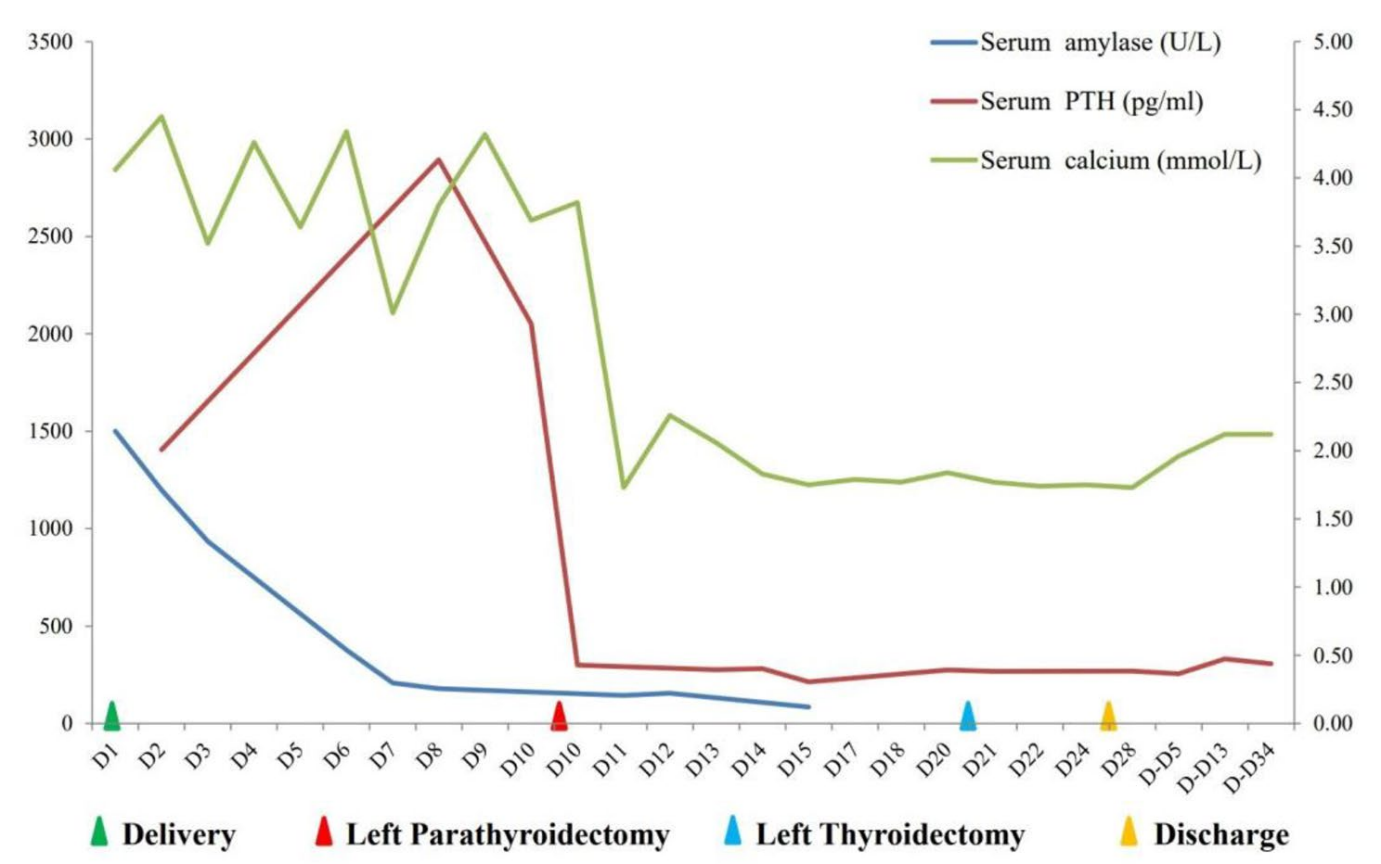
The changes of laboratory indexes before and after treatment

Acute pancreatitis (AP) during pregnancy can arise from a variety of factors, and PHPT is a less common etiology [1, 7]. It has been reported that the prevalence of AP was 6.0%-8.0% among PHPT patients, much higher than that of the general population [8, 9]. Moreover, the PHPT-related AP is more commonly reported in the third trimester (50%) and the second trimester (34.6%) [10]. Parathyroid carcinoma, a rare cause of PHPT, affecting less than 1% of PHPT patients [11]. Based on the published literatures, there were 18 cases of AP due to parathyroid carcinoma, with only two occurring in pregnant women [9, 10, 12–16]. Distinguishing between parathyroid carcinoma and benign adenoma demonstrates a challenge regardless of clinical symptoms and pathological diagnosis. Generally, parathyroid carcinoma is more frequently characterized by renal involvement (56-84% accompanied by nephrolithiasis), high serum calcium levels (>3.5mmol/L), and a significant increase in PTH concentration (exceeding 10 times the upper limit of the reference value) [17–19]. This case was initially thought to be parathyroid adenoma based on the preoperative image screening, leading to a local resection in the first surgery. FNA of tissue was performed and the result needed three days to return. Alternatively, suspected parathyroid tissue can be diluted with saline up to 2 mL and sent for biochemical analysis of PTH [20]. The result will yield within 2 hours. That may be a new way to fast diagnosis. Notably, the patient had undergone cystoscopy two years ago to remove the left ureteral calculi for multiple nephrolithiasis. Her postoperative serum calcium concentration was 3.08mmol/L at that time, but no further examination was conducted. There is no updated data on her serum calcium levels until the onset of AP. She did not receive regular calcium supplements during this pregnancy, possibly due to the known hypercalcemia and nephrolithiasis. The neonatal birth weight, although slightly lower than average, is within normal limits for a baby born at 36^+ 3^ weeks. Figure 2 illustrates the relationship between hyperparathyroidism-dependent hypercalcemia and PTH levels. Serum calcium levels seemed to fluctuate by the PTH curve. PTH stimulates the hydroxylation of 25-OHD, leading to elevated calcitriol levels, which contribute to hypercalciuria and multiple calculus formation [11]. The optimal treatment strategy of parathyroid carcinoma is parathyroidectomy, while the necessity of additional thyroid lobectomy remains debatable [21, 22]. Given that approximately two-thirds of individuals exhibit lymph node or distant metastases at their first presentation, a PET-CT scan was performed [23]. The scan revealed systemic osteoporosis and multi-site calculi, findings not typical for her age. To mitigate the risks associated with a rapid decrease in serum calcium levels, including hypocalcemic symptoms and life-threatening complications, routine calcium supplementation post-parathyroidectomy is essential. Oral calcium supplementation is preferred during rehabilitation, with intravenous calcium infusion as an alternative when necessary. Long-term postoperative follow-ups are crucial for tracking recurrence and metastasis.

In conclusion, this detailed report highlighted a rare case of parathyroid carcinoma-associated pancreatitis in pregnant woman. In this case, prompt diagnosis and urgent delivery via cesarean section were crucial in mitigating potential harm to both the mother and fetus. The key breakthrough was identifying maternal refractory hypercalcemia. It is, therefore, essential to determine serum PTH levels to evaluate the impact of PHPT when persistent hypercalcemia is detected. Surgical interventions are the optimal way to improve prognoses. 

## Data Availability

No datasets were generated or analysed during the current study.
